# Supplementary material for: Do Arabidopsis Squamosa promoter binding Protein‐Like genes act together in plant acclimation to copper or zinc deficiency?
Source: Plant Direct. 2019 Jul 1;3(7):e00150. doi: 10.1002/pld3.150 (PMC6600651; doi:10.1002/pld3.150)
Supplement: Supplementary file 1 [file PLD3-3-e00150-s001.pdf]

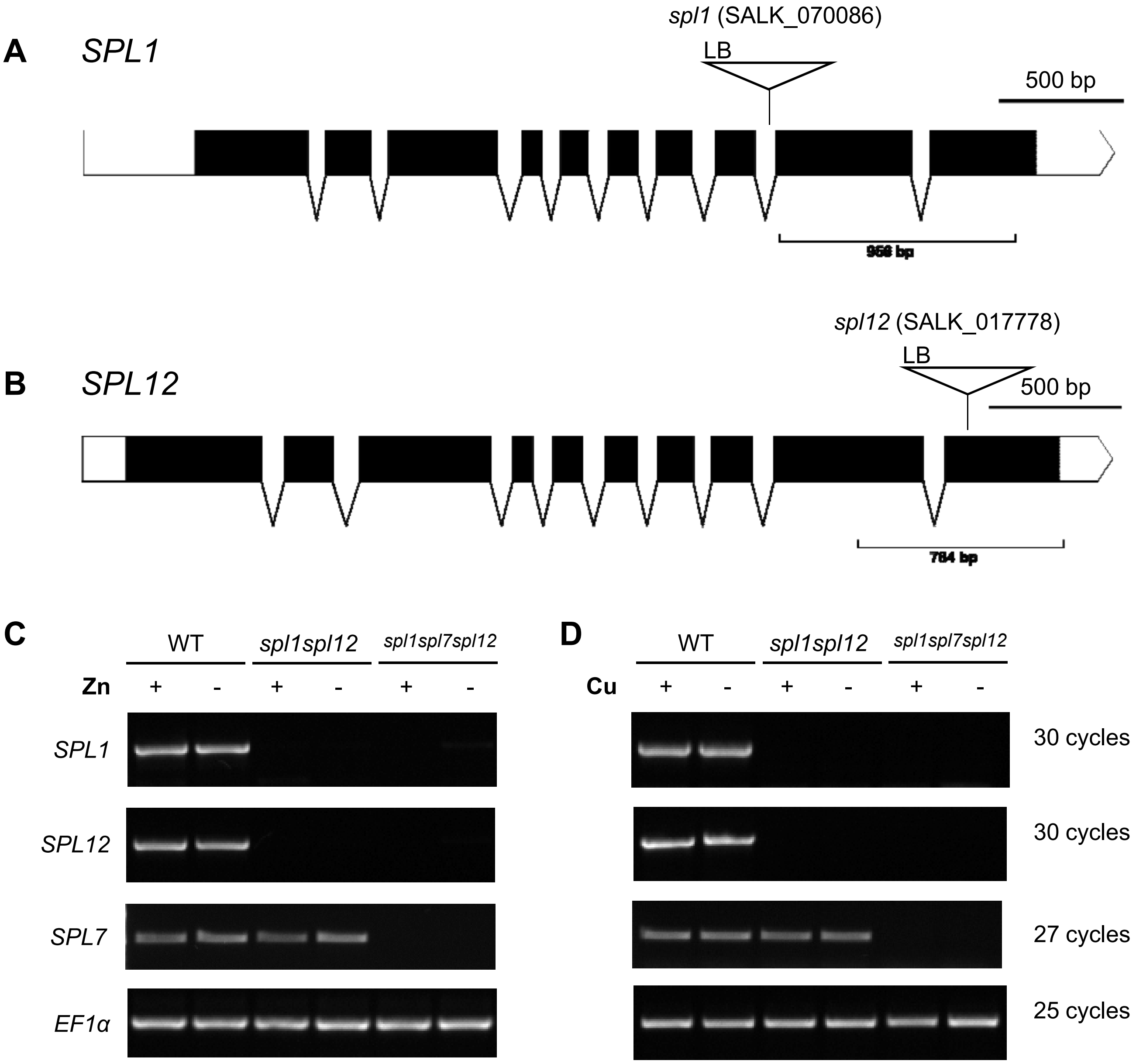

Figure S1: Molecular characterization of *spl1 spl12* and *spl1 spl7 spl12* mutants. (A, B) Schematic representation of the *SPL1* (A) and *SPL12* (B) genomic loci. Black boxes represent exons. The positions of the T-DNA insertions are marked by open triangles, with orientations indicated by marking the positions of left border sequences (LB). Positions and sizes of the fragments amplified in RT-PCRs (see C, D) are marked by thin black lines below. Genomic loci were visualised using the Exon-Intron Graphic Maker (Bhatla, 2012).

(C, D) RT-PCR analysis of *SPL1*, *SPL7* (507 bp) and *SPL12* transcript levels in wild-type, *spl1 spl12* and *spl1 spl7 spl12* mutant seedlings. Total RNA was extracted from 21-day-old seedlings grown either on Zn-sufficient (+, 1 µM $ZnSO_4$) and Zn-deficient (-, 0 µM $ZnSO_4$) agar-solidified media (C) or on Cu-sufficient (+, 0.5 µM $CuSO_4$) and Cu-deficient (-, 0 µM $CuSO_4$) agar-solidified media (D) in short days (11 h). The *EF1α* transcript (fragment of 476 bp) served as a positive control. Note that the reduction of *SPL7* transcript in the *spl7*-2 mutant was confirmed earlier (Bernal *et al.*, 2012). Primer sequences are listed in Supplemental Table 1.

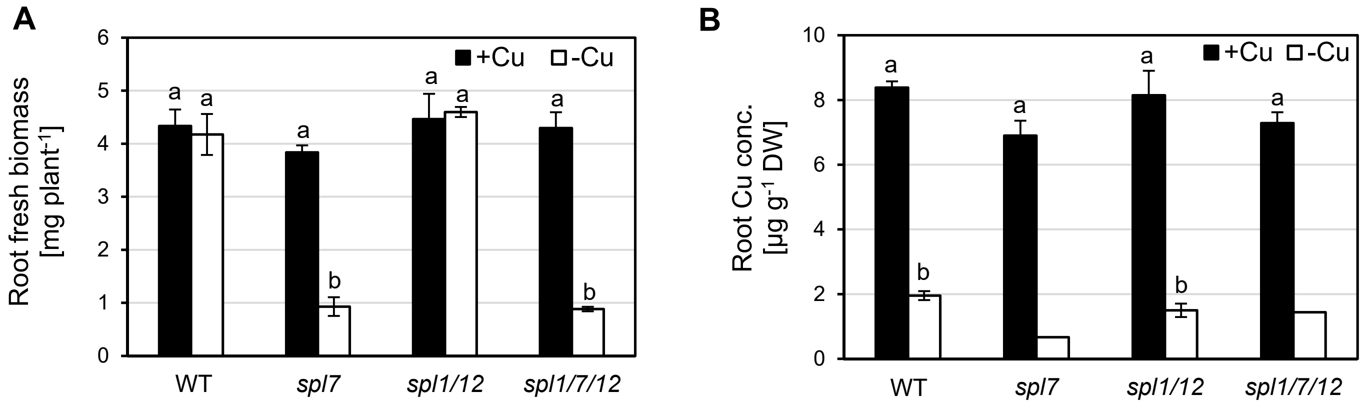

Figure S2: Root biomass and Cu concentrations in wild-type, *spl7*, *spl1 spl12* and *spl1 spl7 spl12* mutant seedlings cultivated on Cu-deficient and -sufficient media. Root biomass (A) and root Cu concentrations (B) of 21-day-old seedlings grown on Cu-sufficient (0.5 µM $CuSO_4$) or Cu-deficient (0 µM $CuSO_4$) agar-solidified media in vertically oriented glass petri plates in short days (11 h). Bars represent arithmetic means ± SD (*n* = 3 replicate plates, each with 20 seedlings). In (B), no SD is shown for *spl7* and *spl1 spl7 spl12* under -Cu in because the dry root biomass was so low that all three replicate plates were pooled to obtain one value. Different characters denote statistically significant differences ($P < 0.05$) between means based on ANOVA (Tukey's HSD). Data are from one experiment representative of three independent experiments.

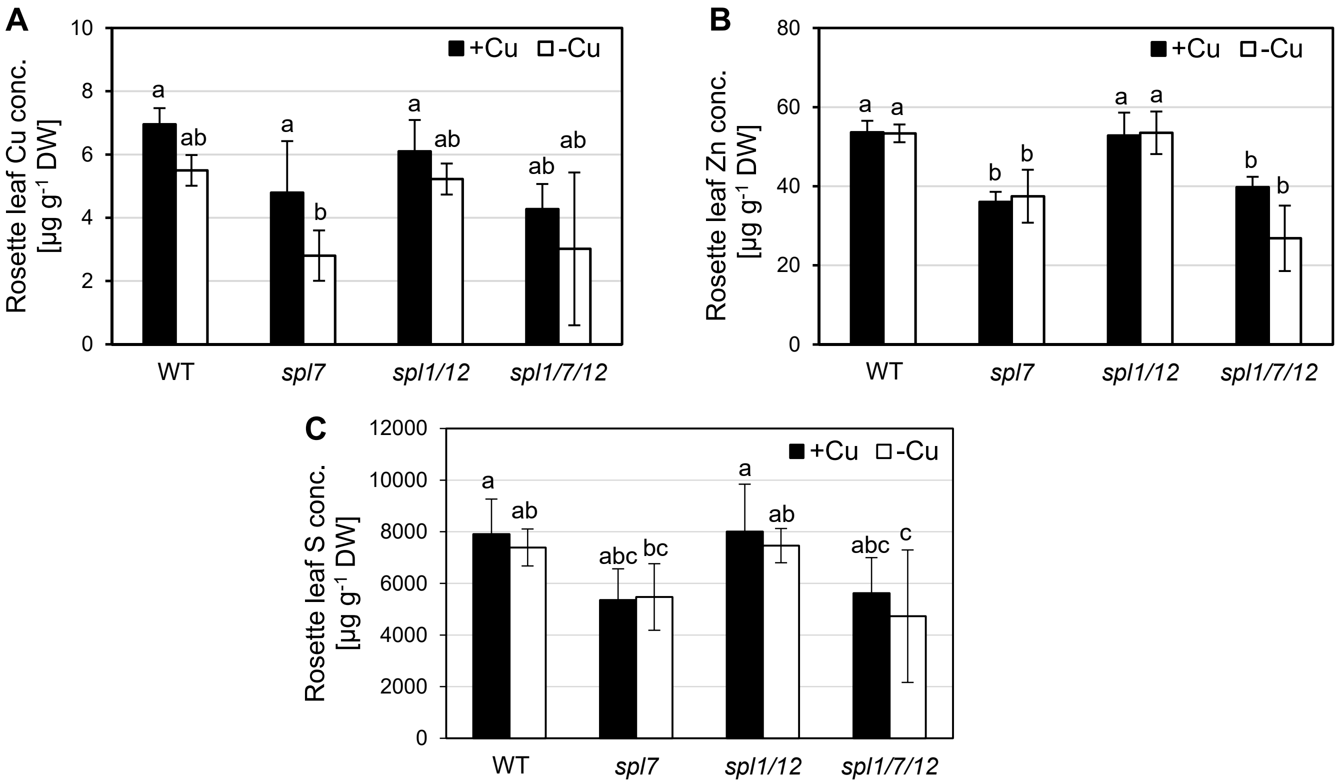

Figure S3: Concentrations of Cu, Zn and S in rosette tissues of reproductive-stage wild-type, *spl7*, *spl1 spl12* and *spl1 spl7 spl12* cultivated on Cu-deficient and -sufficient soil. Cu concentration (A), Zn concentration (B) and S concentration (C) in rosette leaves of 40-day-old plants watered with equal amounts of either tap water or freshly prepared 2 mM $CuSO_4$ in tap water and grown in long days (16 h). Bars represent arithmetic means ± SD (*n* = 6 individual plants per genotype and treatment). Different characters denote statistically significant differences between means based on ANOVA (Tukey's HSD; $P < 0.05$). Data are from one experiment representative of two independent experiments.

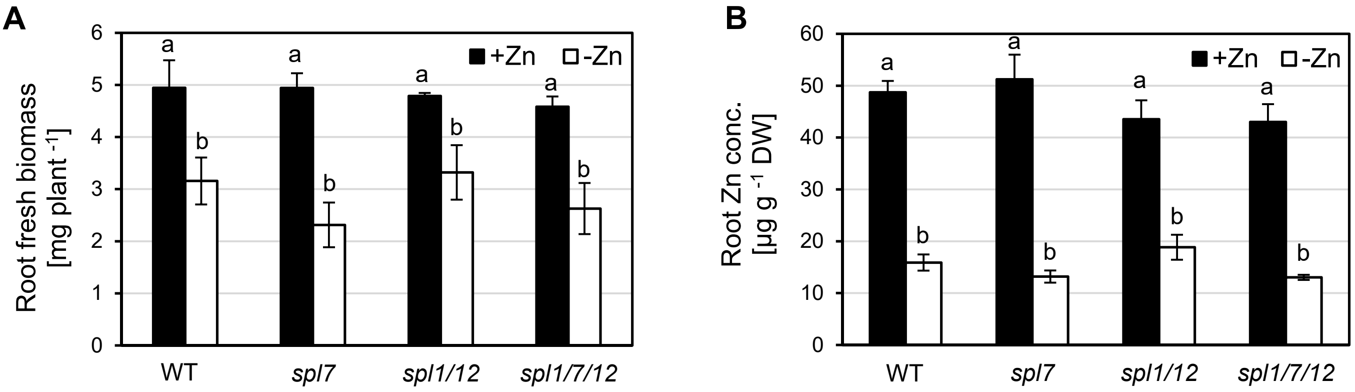

Figure S4: Root biomass of wild-type, *spl7*, *spl1 spl12* and *spl1 spl7 spl12* mutant seedlings cultivated on Zn-deficient and -sufficient media.

(A) Root biomass of 21-day-old seedlings on Zn-sufficient (1 µM $ZnSO_4$) or Zn-deficient (0 µM $ZnSO_4$) agar-solidified media grown in vertically-oriented plastic petri dishes in short days (11 h). Bars show arithmetic means ± SD (*n* = 3 replicate plates, each with 20 seedlings). Different characters denote statistically significant differences between means based on ANOVA (Tukey's HSD; $P < 0.05$). Data are from one experiment representative of three independent experiments.

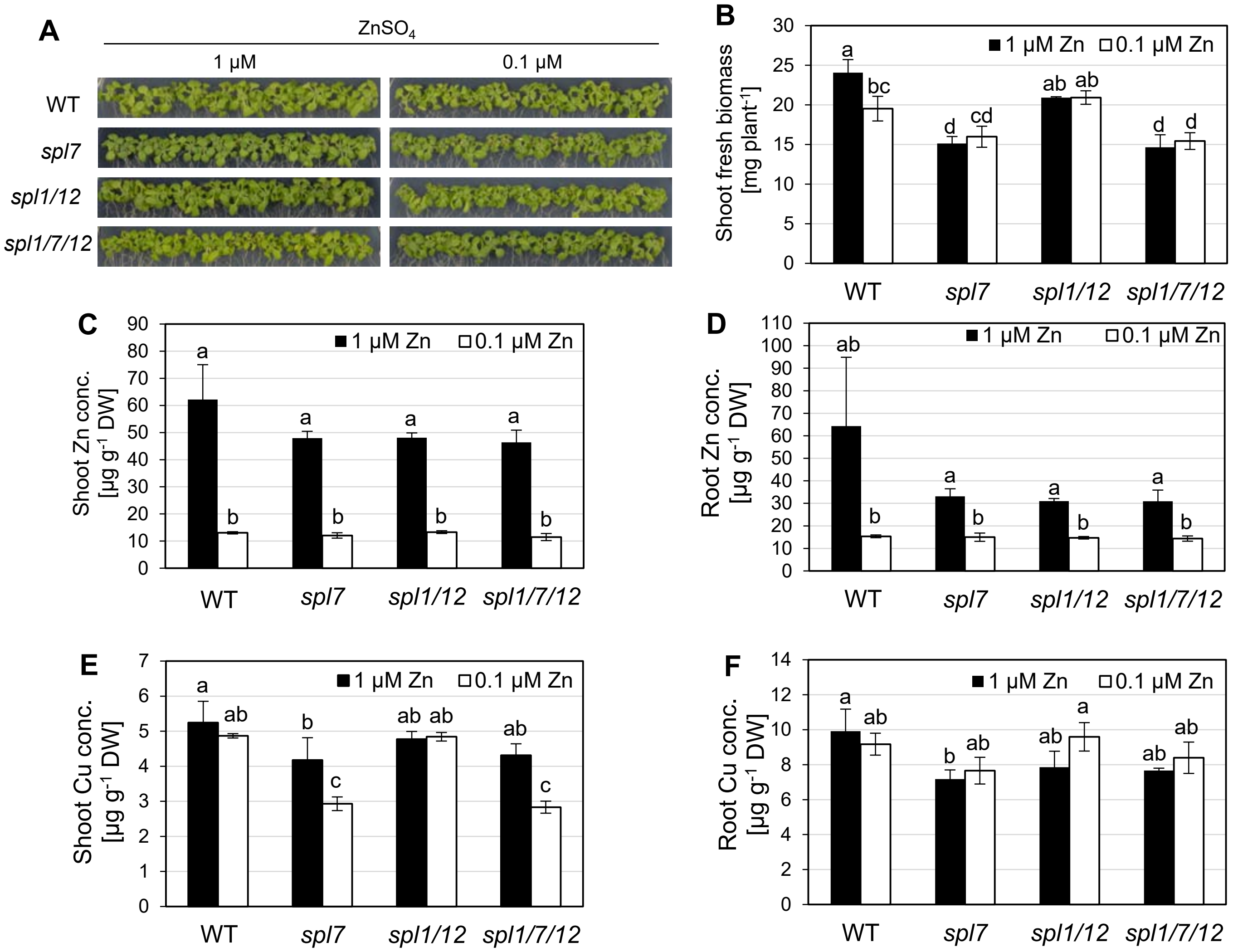

Figure S5: Comparison of zinc deficiency symptoms and tissue Cu levels in wild-type, *spl7* single, *spl1 spl12* double and *spl1 spl7 spl12* triple mutant Arabidopsis seedlings under mild zinc deficiency.

(A) Photographs of 21-day-old seedlings grown on Zn-sufficient (1 µM $ZnSO_4$) or Zn-deficient (0.1 $ZnSO_4$) agar-solidified media in vertically-oriented plastic petri dishes in short days (11 h).

(B) Fresh biomass for shoots of seedlings grown as described in (A). Bars represent arithmetic means ± SD (n = 3 replicate plates, each with 20 seedlings). Different characters denote statistically significant differences ($P < 0.05$) between means based on ANOVA (Tukey's HSD).

(C-F) Zn (C-D) and Cu (E-F) concentrations in shoots (C, E) and roots (D, F) of 21-day old seedlings grown as described for (A). Bars represent arithmetic means ± SD (n = 3 replicate plates, each with 20 seedlings. Different characters denote statistically significant differences ($P < 0.05$) between means based on ANOVA (Tukey's HSD).

Table S1: Oligonucleotides used in this study

Oligo name	Oligo sequence (5' → 3')	Reference
**Oligos used for genotyping & RT-PCR**		
At_spl1_geno_f	CCCCTCTTTGACAATACTCGTC	this work
At_spl1_geno_r	GGATGTATGAGAAGTGACCGCG	this work
At_spl12_geno_f	GGCACTGTTGATCCATCTCCTGATGCTGCG	this work
At_spl7_geno_f	TTGGAAATTCAAGCTGATTCG	this work
At_spl7_geno_r	TCCACCTGTCAAAACCAAGAC	this work
At_spl12_geno_r	GGTATAGGGAAGTTTTACTAGCTTGTTCC	this work
LB_T-DNA	AACGTCCGCAATGTGTTATTAAGTTGTC	Woody et al. 2007
AtSPL1_RT_f	GACTCAGTAGCAGCTTCTTCC	Schwarz 2006
AtSPL1_RT_r	CACACAGACGCAAACCGCAGC	Schwarz 2006
AtSPL7_RT_f	CTATTCTGTTGTACCTGCACCG	this work
AtSPL7_RT_r	CAGTGAACAAGACTGTCTGGC	this work
AtSPL12_RT_f	GGTATAGGGAAGTTTTACTAGCTTGTTCC	Schwarz 2006
AtSPL12_RT_r	GGCACTGTTGATCCATCTCCTGATGCTGCG	Schwarz 2006
AtEF1α_RT_f	TAAGGATGGTCAGACCCGTGA	Sinclair et al. 2018
AtEF1α_RT_r	CAGACTCGTGGTGCATCTCAAC	Sinclair et al. 2018
**Oligos used for qRT-PCR**		
AtEF1α_qRT_f	TGAGCACGCTCTTCTTG	Czechowski et al. 2005
AtEF1α_qRT_r	GGTGGTGGCATCCATCT	Czechowski et al. 2005
AtFSD1_qRT_f	TCGGCTCTTTCCATTGCTT	Bernal et al. 2012
AtFSD1_qRT_r	TGGTCTTCGGTTCTGGAAGTCA	Bernal et al. 2012
AtHEL_qRT_f	CCATTCTACTTTTTGGCGGCT	Talke et al. 2006
AtHEL_qRT_r	TCAATGGTAACTGATCCACTCTGATG	Talke et al. 2006
AtNAS2_qRT_f	CTGACGACGTGGTTAATTCGG	Talke et al. 2006
AtNAS2_qRT_r	TGCCTCGAGCTCCATTTGA	Talke et al. 2006
AtpriMIR398b_qRT_f	CACGAGTAATCAACGGCTGTAATG	Bernal et al. 2012
AtpriMIR398b_qRT_r	TGAGTAAAAGCCAGCCTTGATAAAAG	Bernal et al. 2012
AtZIP9_qRT_f	CCATCACTACTCCGATCGGTGT	Talke et al. 2006
AtZIP9_qRT_r	CACCAATGCTGCAACGCTATAA	Talke et al. 2006

Table S2: AGI locus identifiers of genes mentioned in this article

Gene abbreviation	AGI locus identifier
CITF1	AT1G71200
CSD1	AT1G08830
CSD2	AT2G28190
FSD1	AT4G25100
HMA5	AT1G63440
IRT1	AT4G19690
MIR398b	AT5G14545
MSD1	AT3G10920
NAS2	AT5G56080
PC	AT1G76100, AT1G20340
SPL1	AT2G47070
SPL2	AT5G43270
SPL7	AT5G18830
SPL8	AT1G02065
SPL10	AT1G27370
SPL11	AT1G27360
SPL12	AT3G60030
SPL14	AT1G20980
SPL16	AT1G76580
ZIP9	AT4G33020

Table S3: Composition of soil used for plant cultivation

	Minitray soil new (n = 3)				Minitray soil after 40 days of plant cultivation (n = 7)							
	untreated				watered with tap water				watered with 2 mM $CuSO_4$			
	Extractable		Exchangeable		Extractable		Exchangeable		Extractable		Exchangeable	
**Element**	Mean	SD	Mean	SD	Mean	SD	Mean	SD	Mean	SD	Mean	SD
**Al**	574	436	0.74	0.53	233	35.6	0.75	0.39	344	233	0.88	0.66
**B**	2.19	0.51	0.46	0.03	2.28	0.37	0.41	0.02	2.26	0.28	0.44	0.05
**Ca**	7,990	1260	2,150	878	7,400	1100	2,060	58.5	7,450	1720	1,970	86.9
**Cd**	0.04	0.00	0.02	0.00	0.04	0.01	0.01	0.02	0.04	0.01	n.d.	-
**Cr**	0.11	0.00	0.01	0.00	0.08	0.02	0.02	0.00	0.07	0.02	0.01	0.00
**Cu**	1.31	0.41	0.13	0.03	1.89	0.51	0.03	0.01	124	44.8	0.40	0.10
**Fe**	130	22.7	4.43	0.58	153	13.0	0.82	0.14	154	15.8	0.67	0.26
**K**	219	15.6	200	18.1	212	48.0	159	21.0	239	56.4	153	29.1
**Mg**	878	119	465	53.8	1,570	392	570	67.29	1,520	208	542	92.6
**Mn**	24.0	2.15	2.13	0.41	31.9	3.61	1.52	0.28	30.7	3.31	1.87	0.42
**Mo**	0.02	0.00	0.01	0.00	0.02	n.d.	0.01	0.00	0.02	0.00	0.01	n.d.
**Ni**	0.30	0.10	0.32	0.03	0.25	0.07	0.16	0.10	0.22	0.08	0.12	0.08
**P**	183	5.75	51.7	6.12	135	17.1	13.3	2.13	166	84.0	10.7	3.08
**Pb**	1.28	0.29	0.30	0.04	0.91	0.12	n.d.	-	1.25	0.15	n.d.	-
**S**	-	-	18.1	2.62	-	-	27.3	2.45	-	-	17.8	2.31
**Zn**	4.03	0.34	1.56	0.25	4.06	1.41	0.15	0.03	4.62	1.00	0.09	0.01

Element concentrations given in mg kg^{-1} dry soil; n refers to number of subsamples taken from pooled soil, n.d.: not detectable

Table S4: Relative transcript levels normalized to *HELICASE* (related to Figure 6)
Relative transcript abundance, determined by RT-qPCR, of the Zn deficiency markers *ZIP9* and *NAS2* and the Cu deficiency markers *FSD1*, *CSD2* and *MIR398b* (primary transcript) in 21-day-old seedlings grown on Zn-sufficient (1 µM $ZnSO_4$) or Zn-deficient (0 µM $ZnSO_4$) agar-solidified media in vertically-oriented plastic petri dishes in short days (11 h). Bars represent arithmetic means ± SD (*n* = 2 technical replicates, i.e. independent PCR machine runs, each with three replicate wells per run and transcript).

			ZIP9	***NAS2***	***FSD1***	***MIR398B***	***CSD2***
**WT**	+Zn	Mean	40	180	19,000	310	31,000
		SD	6.4	39	4500	38	3300
	-Zn	Mean	75,000	41,000	1,400	29	1500
		SD	11,000	1,700	78	17	80
spl7	+Zn	Mean	27	300	24	5.1	20,000
		SD	7.3	22	8.2	4.8	670
	-Zn	Mean	78,000	52,000	139	4.1	570
		SD	16,000	2,100	4.2	1.3	28
spl1/12	+Zn	Mean	31	73			
		SD	11	21			
	-Zn	Mean	63,000	41,000			
		SD	1,400	2000			
spl1/7/12	+Zn	Mean	85	150			
		SD	10	17			
	-Zn	Mean	54,000	39,000			
		SD	5,000	790			

Supplemental References

Bernal M, Casero D, Singh V, Wilson GT, Grande A, Yang H, Dodani SC, Pellegrini M, Huijser P, Connolly EL, Merchant SS, Krämer U (2012) Transcriptome sequencing identifies SPL7-regulated copper acquisition genes *FRO4/FRO5* and the copper dependence of iron homeostasis in Arabidopsis. Plant Cell 24 (2): 738-761. doi:10.1105/tpc.111.090431

Bhatla N (2012) Exon-Intron Graphic Maker. http://wormweb.org/exonintron.

Czechowski T, Stitt M, Altmann T, Udvardi MK, Scheible WR (2005) Genome-wide identification and testing of superior reference genes for transcript normalization in Arabidopsis. Plant Physiol 139 (1): 5-17. doi:10.1104/pp.105.063743

Schwarz S (2006) Molecular and Functional Analysis of SBP-Box Transcription Factors in *Arabidopsis thaliana*. PhD thesis, University of Cologne

Sinclair SA, Senger T, Talke IN, Cobbett CS, Haydon MJ, Krämer U (2018) Systemic Upregulation of MTP2- and HMA2-Mediated Zn Partitioning to the Shoot Supplements Local Zn Deficiency Responses. The Plant Cell 30 (10): 2463-2479. doi:10.1105/tpc.18.00207

Talke IN, Hanikenne M, Krämer U (2006) Zinc-dependent global transcriptional control, transcriptional deregulation, and higher gene copy number for genes in metal homeostasis of the hyperaccumulator *Arabidopsis halleri*. Plant Physiol 142 (1): 148-167. doi:10.1104/pp.105.076232

Woody ST, Austin-Phillips S, Amasino RM, Krysan PJ (2007) The WiscDsLox T-DNA collection: an Arabidopsis community resource generated by using an improved high-throughput T-DNA sequencing pipeline. Journal of Plant Research 120 (1): 157-165. doi:10.1007/s10265-006-0048-x
