# Supplementary material for: Do Arabidopsis Squamosa promoter binding Protein‐Like genes act together in plant acclimation to copper or zinc deficiency?
Source: Plant Direct. 2019 Jul 1;3(7):e00150. doi: 10.1002/pld3.150 (PMC6600651; doi:10.1002/pld3.150)
Supplement: Supplementary file 2 [file PLD3-3-e00150-s002.pdf]

Response to reviewers – Schulten et al. (**2019-00273**)

Dear Ivan,
We thank all reviewers for their careful reading of the manuscript and thoughtful comments and you for handling this manuscript. Point-by-point responses are listed below.

With kind regards,
Ute

Editor's comments:
The two critiques that need to be addressed are in reference to our requirement that the results support the conclusions.

1. As reviewer #1 suggests, it is important to demonstrate that the soil is creating Cu efficient conditions.

   Our RESPONSE: See Reviewer #1, 1.

2. … to address is that the 0 Zn plants are so sick that it is hard to make conclusions about their Cu status.

   Our RESPONSE: See Reviewer #3,

Reviewer #1:

1. For the soil-based experiments there is no indication of the Cu concentration in the soil used. The only indication that the soil is low in Cu is spl7 phenotype, which might not be sufficient to claim that these plants experienced Cu deficiency. Moreover observed phenotype might be also an effect of other nutrient deficiencies. I think that the data on the chemical composition of the soil used should be provided.

   Our RESPONSE: We did not provide any measurements of soil nutrient contents in our first submission because there is no knowledge basis presently for predicting from such measurements plant nutrient status or even nutrient bioavailabilities. In our opinion, Cu concentrations in plant tissues allows a much better-founded estimate of whether a (wild-type) plant is experiencing Cu deficiency or not. When supplemented with 2 mM $CuSO_4$, the *spl7* mutant was much healthier (flower health, plant size) compared to our unsupplemented soil, indicating that either Cu or sulfur deficiency is causal of the phenotypic differences. As provided in Supplemental Figure S3A, Cu concentration in *spl7* grown in unsupplemented soil drops well below the established critical deficiency limit of 5 µg/g DW, whereas the sulfur concentrations were in the range of plant requirements for optimal growth as defined by Marschner (1995). Therefore, we concluded that plants experience Cu deficiency in our unsupplemented soil which can be alleviated by watering additional Cu.

   CHANGES made:

- We are including in a new panel total S concentration in rosette leaves (Fig. S3C), in addition to Cu and Zn.
- To follow the request of reviewer #1, we have added a Supplemental Table (Table S3) of nutrient contents in our untreated Minitray soil for reference.
- We have expanded the Methods to incorporate multi-element analysis of soils.
- We have made small additions to Results (3.2) to refer to these new supplemental display items.

2. Methods used are in several cases not described in sufficient way. Instead authors refer to published works, but not all of them are open access. Therefore I would suggest to describe used methods with more details.

   Our RESPONSE: See below.

   CHANGES made:

- We added a sentence in methods (Growth conditions) specifying the mineral nutrient composition of our modified Hoagland's medium
- We added text in section "Determination…" specifying methods used for the digestion of plant material and multi-element analysis
- We added text in section "Measurement…" specifying how tissues were homogenized and extracted, as well as all details for chlorophyll quantification.
- We added text in "RNA…" specifying how cDNAs were diluted, the precise composition and final volume as well as thermal cycling conditions of qPCR reactions.
- We added text in "In-gel…" describing also each step of what was published in Beauchamp and Fridovich. 1971.

3. Fig 3B. There is only one plant for *spl7* on -Cu conditions. I understand that this is caused by the fact that very few of the *spl7* plants produced siliques. However, it is mentioned that 2 independent biological experiments were performed. I think that statistical analysis would be stronger if the 2 experiments would be pooled together, and data were fitted the linear model that would use experiment as a factor. This could make the statement about the decreased number of siliques per plant in *spl7* line stronger.

   Our RESPONSE:

   Thank you for pointing this out to us. For this panel, we corrected our statistics. Five of the plants produced zero siliques and only a single plant produced any siliques at all. In the second independent experiment, we observed this again. Therefore, these five plants producing zero siliques must also go into the statistical analysis for this figure.

   CHANGES made:

   The statistical analysis for Fig. 3B was repeated incorporating also the plants producing no siliques (= 0).

4. If the Hoagland solution was depleted from $CuSO_4$ x 5 H_2O that it typically contains, was the sulphur content adjusted. Would it be necessary to do or not? (Minor comment)

   Our RESPONSE:

   The sulfur content in the Hoagland solution was not adjusted when leaving out $CuSO_4$ because we do not believe that it is necessary to do so. With a final concentration of 0.5 µM $CuSO_4$, the contribution to the total sulfur content is minor compared to the sulfur provided in the form of $MgSO_4$ (750 µM final concentration) in our modified Hoagland's medium.

   CHANGES made: none.

5. For the measurements of chlorophyll, which wavelength was used? Number of the replicates is not mentioned. (Minor comment)

   Our RESPONSE:

   The wavelengths for chlorophyll measurements were as detailed in Porra *et al.* (1989). We stated the exact number of replicates in the figure legends for each graph.

   Changes made:

- We are now providing the wavelengths and the formula used for calculation of chlorophyll contents in the Methods (section "Measurement…"; see also our response to reviewer #1, comment 2 above)

6. (a) In the "In-gel detection of superoxide dismutase activities" paragraph it should be mentioned how many plants per conditions were tested. (b) Moreover it is stated: "Specific inhibitors were used during the activity staining period in replicate gels (data not shown) for the identification of different SOD isoforms". If the data exist why not show them? If the specificity was checked the result should be mentioned. (Minor comment)

   Our RESPONSE:

   (a) Generally, we state the information on experimental repeats, within-experiment replication and the number of seedlings pooled per extraction in the figure legends. Thus, we added the missing information there (see Changes below).

   (b) We are sorry that we did not reflect this aspect correctly in the Methods text because of poor memory: When checking our lab books again, we found that specific inhibitors were used for the initial set-up during the establishment of this method for this specific project. Our data agreed with published data (Kliebenstein et al., 1998). The positions of bands for individual SOD isoforms never change in our hands.

   CHANGES made:

   (a) We modified the legend texts of Fig. 1Eand Fig. 5C by adding the number of seedlings that were pooled (and from how many replicate plates) per extract.
   (b) We modified the Methods text to more accurately reflect what was actually done exactly (see also 2. above)

7. Figures: Fig.1 A/D Is this one biological experiment? Was it replicated? (Minor comment)

   Our RESPONSE:

   The final sentence on the experiment replication ("Data are from one experiment representative of two to three independent experiments.") applies to the entire figure and not just to Fig. 1E.

   CHANGES made:

   We modified the figure legend of Fig. 1: "All Data in Fig. 1 are from one …"

8. Fig 1C. Abbreviations FW, DW should be explained in the caption.

   Our RESPONSE: See below.

   CHANGES made: Abbreviations are now defined in this figure legend.

9. Fig. 1 E. How was the activity of these proteins detected? The size of the proteins detected should be included here (Similar for the Fig. 5B)

   Our RESPONSE: Since the in-gel staining for SOD activity is performed in native gels as specified in the respective figure legends, proteins do not just run according to size but also according to electric charge. For example, FSD1 (23.7 kDa) and CSD2 (22 kDa) run very differently in the native gels despite their similar protein sizes. Therefore, it does not appear useful to us to mark protein sizes in the gel pictures.

   CHANGES made: We added methodological details including the fact that we ran native gels in the Methods (see also 2. and 6. above)

10. Fig 2. It should be mentioned in the text how were the aborted siliques defined and what distinguished them form normal ones.

    Our RESPONSE: Aborted siliques were defined as siliques that did not elongate to a size >10 mm and did not form seeds within the silique.

    CHANGES made: Addition in legend of Figure 2, after "White arrows highlight positions of aborted siliques", "(i.e., max. length < 10 mm and no seed filling)."

11. Fig 3. For the number of seeds per silique - were these seeds all viable?

    Our RESPONSE: The seeds we counted in each silique appeared uniform, mature and normally developed. We never observed evident changes in viability or germination rate in any of the genotypes studied here. If siliques were produced as defined above they did contain viable seeds.

    CHANGES made: We inserted in legend of Fig. 3C, before "Different characters…": "The seeds we counted in mature, manually opened siliques were brown-colored and of uniform size and appearance. "

12. Fig 6. It should be mentioned which tissue is RNA coming from, n.d should be explained

Our RESPONSE: See below.
CHANGES made: Legend of Figure 6 now contains type of tissue and the definition of n. d.

13. Fig6 B why is there only spl7 checked?

    Our RESPONSE: *FSD1* and *MIR389b* are known as direct targets of SPL7 under Cu deficiency. Since we had no indication for a role of SPL1 and SPL12 similar to SPL7 under Cu deficiency from any of our other data in this manuscript, we only determined transcript levels in the wild type and in the *spl7* mutant to investigate whether SPL7 targets respond to Zn deficiency in an SPL7-dependent manner. This hypothesis was based on a function for the Chlamydomonas Cu response regulator CRR1 under Zn deficiency.

    CHANGES made: None.

14. Figures 4 and 5 could be easily merged in one.

    Our RESPONSE: We agree that this could easily be done. However, we prefer to keep these figures separate as they are making two different points: Fig. 4 addresses general phenotypes under Zn deficiency whereas Fig. 5 specifically addresses Cu accumulation and Cu status markers under Zn deficiency.

15. Abstract could be written shorter. There are information that are not indispensable to understand the message of the manuscript (ex. : which additionally comprises a second group of small SPL proteins targeted by miRNA156 with roles in plant development.")

    Our RESPONSE: Our abstract conforms to the instructions. Since the short SPL proteins are more widely known than SPL7 or other long SPLs, we would like to enable readers who know the short SPL transcription factors to correctly understand what we worked on.

16. In the abstract it is mentioned: "We observed that CuZnSOD activity is strongly downregulated in Zn-deficient *A. thaliana*, in association with an about 94% reduction in the abundance of the CDS2 transcript, a known target of miR398."
    Shouldn't it be CSD2 transcript?

    CHANGES made: We corrected the typo. It now reads "*CSD2*" transcript

17. Results:
    "The previously described growth defects of the *spl7* single mutant under Cu deficiency were evident through strongly reduced fresh biomass and lower chlorophyll concentrations." There should be reference included.

    CHANGES made: Reference to Bernal et al. 2012 was added here.

18. "... the *spl1 spl7 spl12* triple mutant mimicked *spl7* whereas the *spl1 spl12* mutant performed like the wild type in media lacking added" I suggest being more precise and mention which particular phenotypes this refers to.

    Our RESPONSE: Thank you for alerting us to the fact that this is unclear.

CHANGES made: The corresponding sentence was changed to read: "When cultivated on medium lacking added Cu, the *spl1 spl7 spl12* triple mutant mimicked *spl7* in terms of seedling appearance, fresh biomass and chlorophyll concentrations, whereas the *spl1 spl12* mutant was indistinguishable from the wild type (Fig. 1A-C, Fig. S2)."

19. Whenever mentioning CRR, adding that it is Chlamydomonas gene would make it more precise.

    CHANGES made: The abbreviation Cr was added to CRR1 to make this more precise.

20. Paragraph 3.2 "Compared to the wild type, rosette Zn concentrations were generally slightly decreased by about 30% to ca. 35 µg g-1 DW in *spl7* and the triple mutant." Mentioning Zn here is a bit out of the blue- I suggest adding one sentence justifying the link of SPL7 with Cu and Zn homeostasis.

CHANGES made: Because of an addition made to the preceding sentence in response to Reviewer #1, 1., we are now introducing this statement in the text.

Reviewer #2:

1. Include one or two more internal control genes for qRT-PCR.

Our RESPONSE: We are aware that this has been suggested as a general standard. However, we find that depending on whether we compare tissues or treatments different constitutively expressed reference genes are expressed more uniformly. Also, constitutively expressed reference genes of which transcript levels differ strongly from target genes are non-optimal to use for reference. Therefore, we are convinced that our approach is the most accurate.

CHANGES made: We added data evaluated relative to *HELICASE* as a reference gene in the supplement to satisfy the reviewer (Table S4; now referred to in legend of Fig. 6).

2. In results under 3.2, the seed number per silique was reduced in spl7 as well as in the double spl1 spl12 mutant as compared to WT both in +Cu and –Cu, but in the triple mutant of spl1,7,12 the number of seeds further decreased specially in -Cu condition. Authors have suggested a probable role of SPL1 and SPL12 in Cu assimilation in absence of SPL7. However, no other results are supporting this. This should not be correlated with Cu acclimation but with seed development. Else this line can be removed or should be reworded for more specific and precise meaning.

Our RESPONSE: We regret we do not entirely understand this comment (especially the final two sentences).

CHANGES made: We rephrased the corresponding sentence, to read as follows: "Under -Cu conditions, the difference was even more pronounced. This is an initial observation consistent with a small contribution of *SPL1*/*SPL12* to seed development under low Cu conditions. Additional experiments will be necessary in the future to confirm and examine this further. "

Reviewer #3:

1. The presented results are generally negative, but it is also difficult to eliminate any functional overlap.

   Our RESPONSE: We agree with the reviewer on this general and fundamental difficulty associated with negative results.

   CHANGES made: We added one sentence in the discussion at the end of 4.1: "In summary, we did not obtain clear evidence for a functional overlap between *SPL7* and *SPL1/SPL12* under the conditions employed and considering the phenotypic parameters examined in this study. It appeared that only when *spl7* plants suffered severe physiological Cu deficiency under Cu-deficient growth conditions, *SPL1/SPL12* may act to slightly alleviate the severity of some defects in *spl7* mutants (see Figs. 1C and 3C). These observations will require further study in the future, and they may not be physiologically relevant for wild-type plants. "

2. The -Zn plants are very unhealthy and we may not be able to evaluate the physiological response (Figs. 4/5).

   Changes made:

   - We added to the supplement an additional experiment in which we implemented milder Zn deficiency treatments and obtained data analogous to Figs. 4 and 5. Based on these additional data (Fig. S5), the conclusions of from the first submission remain entirely unchanged.
   - We added a sentence in results (3.3): "We confirmed these observations in an experiment in which we implemented milder Zn deficiency conditions (Fig. S5)."

3. Figure 2. The difference between O and P should be explained in the legend.
   Our RESPONSE: Thank you for pointing this out.
   Changes made: As addition to the figure legend of Fig. 2: "(O) and (P) show flowers on a lateral inflorescence stem and the main inflorescence stem, respectively."
